# Combining Regional Energy and Intuitionistic Fuzzy Sets for Infrared and Visible Image Fusion

**DOI:** 10.3390/s21237813

**Published:** 2021-11-24

**Authors:** Xiaoxue Xing, Cong Luo, Jian Zhou, Minghan Yan, Cheng Liu, Tingfa Xu

**Affiliations:** 1 College of Electronic Information Engineering, Changchun University, Changchun 130012, China; 190401069@mails.ccu.edu.cn (C.L.); 200401085@mails.ccu.edu.cn (J.Z.); 200401084@mails.ccu.edu.cn (M.Y.); 210401102@mails.ccu.edu.cn (C.L.); 2 School of Optics and Photonics, Beijing Institute of Technology, Beijing 100081, China; ciom_xtf1@bit.edu.cn

**Keywords:** infrared and visible images, images fusion, RE, IFS, NSST

## Abstract

To get more obvious target information and more texture features, a new fusion method for the infrared (IR) and visible (VIS) images combining regional energy (RE) and intuitionistic fuzzy sets (IFS) is proposed, and this method can be described by several steps as follows. Firstly, the IR and VIS images are decomposed into low- and high-frequency sub-bands by non-subsampled shearlet transform (NSST). Secondly, RE-based fusion rule is used to obtain the low-frequency pre-fusion image, which allows the important target information preserved in the resulting image. Based on the pre-fusion image, the IFS-based fusion rule is introduced to achieve the final low-frequency image, which enables more important texture information transferred to the resulting image. Thirdly, the ‘max-absolute’ fusion rule is adopted to fuse high-frequency sub-bands. Finally, the fused image is reconstructed by inverse NSST. The TNO and RoadScene datasets are used to evaluate the proposed method. The simulation results demonstrate that the fused images of the proposed method have more obvious targets, higher contrast, more plentiful detailed information, and local features. Qualitative and quantitative analysis results show that the presented method is superior to the other nine advanced fusion methods.

## 1. Introduction

Infrared (IR) and visible (VIS) images fusion focuses on synthesizing multiple images into one comprehensive image, which can be applied in face recognition [1], target detection [2], images enhancement [3], medicine field [4], remote sensing [5], and so on. The source images applied in image fusion come from different sensors. The IR sensor can capture the heat information radiated by objects. IR images have a low spatial resolution, less background information, poor imaging performance, and high contrast pixel intensities. In contrast, the VIS images provide abundant background, rich detailed texture information, and a high spatial resolution. Hence, the effective fusion of the two types of images will provide more useful information and better human visual effects, and that is beneficial for the subsequent research work [6,7].

The selection of the fusion rule is very crucial, and it decides the fusion effects. The essence of image fusion is how to reasonably choose the valuable pixels of multiple source images and integrate them into one image. Image fusion can be considered as the transfer of image information. The process is actually a many-to-one mapping, which contains strong uncertainty. To solve the problem, the energy-based fusion strategy is often used to enhance the image quality and reduce the uncertainty. Zhang [8] has presented a RE-based fusion rule for the IR and VIS image fusion which can preserve more prominent infrared targets information. Srivastava [9] has proposed a local energy-based method to fuse the multi-modal medical images that can obtain better fusion performance. Liu [10] has presented the average-RE fusion rule to fuse the multi-focus and medical images, and the results show that the fused image contains more information and edge details. Thanks to the consideration of the correlation of the pixels, the energy-based fusion strategies can overcome the uncertainty of improper pixels selection and improve the quality of the fused image to some extent.

In image fusion, the possibility that uncertainty and ambiguity occur can be considered extremely likely (due to sampling techniques, noising, blurring edges,...). Therefore, it is imperative the implementation of adaptive items to manipulate data uncertainty. Scientific research has produced a lot of good results by fuzzy logic and techniques. Versaci proposed a fuzzy geometrical approach to control the uncertainty of the image [11]. As the extension of the fuzzy sets (FS) theory, intuitionistic fuzzy sets (IFS) is described by membership, non-membership, and hesitation degrees which are more flexible and practical than FS in dealing with fuzziness and uncertainty [12]. In recent years, many relative methods have been developed in the field of image fusion. T. Tirupal [13] has presented Sugeno’s intuitionistic fuzzy set (SIFS) -based method to fuse multi-modal medical images. The image obtained by this algorithm can distinguish the edge of soft tissue and blood vessel clearly, which is helpful for case diagnosis. C. H. Seng [14] has proposed a method based on probabilistic fuzzy logic to fuse through-the-wall radar images, and the results show that fused image has a higher contrast to help improve the detection rate of the target. Zhang [12] has designed a method based on fractional-order derivative and IFS for multi-focus image fusion, and the results show that the method can avoid the artifacts and preserve the detailed information. It can be proved that IFS can solve the problems that existed in the image fusion process, which is suitable for image fusion.

In this paper, we combine RE and IFS to design a new image fusion strategy to enhance the fused image quality. To better extract the detailed features, we use non-subsampled shearlet transform (NSST) to decompose the IR and VIS images to get low- and high-frequency sub-bands. For the high-frequency sub-bands, the ‘max-absolute’ rule is adopted to obtain the fused detailed information. For the low-frequency sub-bands, the new fusion strategy is implemented to achieve the fused low-frequency components, and the strategy can be described by two steps as follows. Firstly, the RE-based fusion rule is performed on the low-frequency layers to get a pre-fusion image, which allows more target information preserved in the resulting image. Then, the IFS is introduced to obtain the final fused images, which enables more texture information to be transferred to the resulting image. We use the inverse NSST to reconstruct the final fused result. Simulation experiments on the public datasets demonstrate that this method outperforms other advanced fusion methods. The fused images have better stable quality, more obvious targets, higher contrast, more plentiful detailed information, and local features.

The rest of the paper is arranged as follows. The basic principle of NSST and fuzzy theory are reviewed in Section 2. The fusion rules proposed in this study are introduced in Section 3. Experiments and results analysis are presented in Section 4. The paper is summarized in Section 5.

## 2. Related Works

### 2.1. Basic Principle of NSST

The fusion methods based on multi-scale geometric analysis (MGA) are widely used in IR and VIS image fusion [15,16,17,18,19,20,21,22,23]. MGA tools can represent the images at different scales and different directions, and these characteristics are helpful to extract more detailed information of the images. Among these MGA tools, NSST is regarded as the most popular one [24]. Many researchers have proved that the fused images of NSST-based method are more suitable for the human visual system. NSST is proposed by K. Guo and G. Easley et al. [25,26], and the model of NSST can be described as follows.

Assume *n* = 2, the affine systems with composite dilations are the collections of the form [25]:

(1) $$\psi_{AB}\left(\psi \right)=\left\{\psi_{j,l,k}\left(x\right)={\left|\det A\right|}^{\frac{j}{2}}\psi \left(B^{l}A^{j}x-k\right):j,l\in Z,K\in Z^{2}\right\}$$

where $\psi \in L^{2}\left(R^{2}\right)$, A is a 2 × 2 invertible matrix, so is B. By choosing ψ, A, and B appropriately, we can make $\psi_{AB}$ an orthonormal basis or, more generally, a Parseval frame (PF) for $L^{2}\left(R^{2}\right)$. Typically, the members of B are shear matrices (all eigenvalues are one), while the members of A are matrices expanding or contracting on a proper subspace of $R^{2}$. These wavelets are of interest in applications because of their tendency to produce “long, narrow” window functions well suited to edge detection.

Assume $A_{a}=\left[\begin{array}{cc} a & 0\\ 0 & \sqrt{a} \end{array}\right]$, $B_{s}=\left[\begin{array}{cc} 1 & s\\ 0 & 1 \end{array}\right]$, the shearlet system is shown as Equation (2), $\psi_{ast}\left(x\right)$ is a shearlet [26]. Shearlet can be considered as a special example of composite wavelets in $L^{2}\left(R^{2}\right)$, whose elements range not only at various scales and locations, like wavelets, but also at various orientations [27].

(2) $$\psi_{ast}\left(x\right)=\left\{a^{-\frac{3}{4}}\psi \left(A_{a}^{-1}B_{s}^{-1}x-t\right),a\in R^{+},s\in R,t\in R^{2}\right\}$$

**Figure 1:**
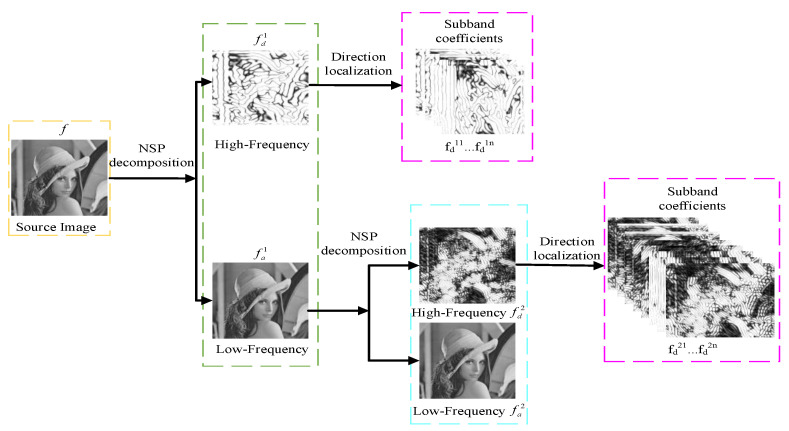
NSST decomposition structure with two levels.

Figure 1 shows the NSST decomposition structure of two levels. The source image *f* is decomposed into a low-pass image $f_{a}^{1}$ and a band-pass image $f_{d}^{1}$ by a non-subsampled pyramid (NSP). After that, the NSP decomposition of each layer is iterated on the low-frequency component obtained from the upper layer decomposition. The shearlet filter banks are used to decompose $f_{d}^{1}$ and $f_{d}^{2}$ to attain the high-frequency sub-bands coefficients.

### 2.2. Fuzzy Set Theory

Zadeh presented the fuzzy set (FS) theory in 1965 [27]. According to the FS theory, the membership degree is used to quantify the uncertain information expressed by the interval [0,1]. A value between 0 and 1 is used to represent the membership degree. The value of 0 means the non-membership, and the value of 1 means the full membership. The sum of the element membership degree is 1.

FS theory is good at representing qualitative knowledge with unclear boundaries, which plays a vital role in eliminating the vagueness that existed in images [28]. Many studies show that the image fusion methods based on FS theory are superior to other conventional algorithm models. The composite methods that combine the FS theory with other representation methods can strictly select reliable pixel information of source images [29].

According to general set theory, there is a relationship of belonging to or not belonging between elements and sets. Let U denote the universe, u∈U, A⊆U, and the characteristic function $\chi_{A}$ of *A* is defined as follows [30]:

(3) $$\begin{array}{c} \chi_{A}:U\to \left\{0,1\right\}\\ u\to \chi_{A}\left(u\right)\in \left\{0,1\right\} \end{array}\}$$

(4) $$\chi_{A}=\{\begin{array}{cc} 1, & u\in A\\ 0, & u\notin A \end{array}$$

In FS theory, the definition of membership function evolves from the characteristic function in general set theory. Let $\underline{A}$ denote a fuzzy subset of U, and the membership function $\mu_{\underline{A}}$ can be defined as follows [30]:

(5) $$\begin{array}{c} u_{\underline{A}}:U\to \left[0,1\right]\\ u\to \mu \underline{{}_{A}}\left(u\right)\in \left[0,1\right] \end{array}\}$$

It can be seen from Equation (5), the FS theory is established based on the membership function. Therefore, the membership function is very important in fuzzy mathematics.

The image with the resolution of M×N can be seen as a fuzzy pixel set, as follows [30]:

(6) $$X=U_{i=1}^{M}U_{j=1}^{N}\frac{u_{ij}}{x_{ij}}$$

where $x_{ij}$ means the grayscale value of the pixel (i,j). $\mu_{ij}$ belongs to [0, 1], which represents the membership degree of the pixel (i,j). $\left\{\mu_{ij}\right\}$ represents the fuzzy characteristics plane, which is composed of all $\mu_{ij}$. $\mu_{ij}$ is calculated by the membership degree function. Different membership degree functions can obtain different $\mu_{ij}$. Therefore, it is convenient to adjust the $\mu_{ij}$ to acquire different enhancement effects.

## 3. Proposed Method

**Figure 2:**
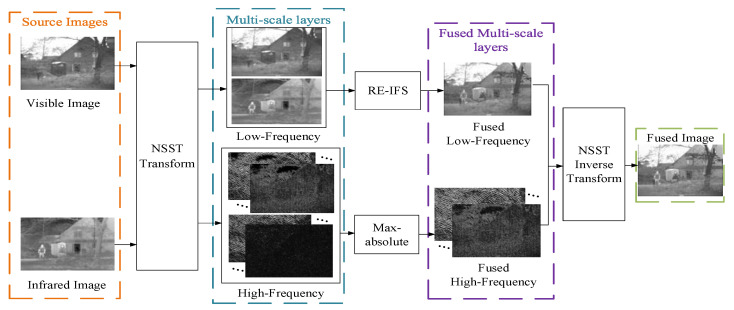
The overall framework of the proposed algorithm.

In this section, we propose a new fusion strategy for IR and VIS images: a combining RE and IFS method in NSST domain (RE-IFS-NSST). Figure 2 illustrates the overall framework of the algorithm. The fusion process can be mainly divided into 4 parts: NSST decomposition, the low-frequency sub-bands fusion, the high-frequency sub-bands fusion, and the NSST reconstruction.

### 3.1. NSST Decomposition

In this paper, the NSST is used to decompose the source images. The IR and VIS images are decomposed by NSST to obtain high- and low-frequency sub-bands according to the Equations (7) and (8). The $IR_{L}$ and $VIS_{L}$ are the low-frequency sub-bands of the IR and VIS images, respectively; the $IR_{H}^{j,k}$ and $VIS_{H}^{j,k}$ are the high-frequency sub-bands of the IR and VIS images at the *j* level with the *k* direction, respectively.

(7) $$\left\{IR_{L},IR_{H}^{j,k}\right\}=NSST\_DE(IR)$$

(8) $$\left\{VIS_{L},VIS_{H}^{j,k}\right\}=NSST\_DE(VIS)$$

where the NSST_DE(·) represents the NSST decomposition function of the input image.

### 3.2. The Rule for Low-Frequency Components

The low-frequency components contain most of the energy information such as contour and background information [31]. In this paper, the RE and IFS are used to construct the fusion rule for the low-frequency components. The method consists of two steps: (1) the pre-fusion based on RE; (2) the final fusion based on IFS.

In the low-frequency of IR images, the salient targets are usually located in regions that have large energy. The fusion rule based on RE can transmit the energy information of the IR images to the fused image which can achieve better performance on the extraction of the target information. Therefore, we firstly adopt RE-based fusion rule to get the pre-fusion image. Based on the pre-fusion image, we secondly adopt the IFS-based method to get the final result. IFS can be described by membership, non-membership, and hesitation degrees at the same time. In accordance with the membership degree, the pixels of the source images can be easily, precisely, and effectively classified into targets and background information. By means of the IFS-based method, the texture information of VIS images can be transferred to the resulting image.

(1) The pre-fusion based on RE

$IR_{L}$ and $VIS_{L}$ represents the low-frequency components of IR and VIS images, respectively. $IR_{L}$ and $VIS_{L}$ are firstly fused based on the RE-based fusion rule to achieve the pre-fused low-frequency image. The RE is calculated as follows [32]:

(9) $$E_{S}\left(m,n\right)=\sum_{\left(i,j\right)\in \Omega \left(m,n\right)}A_{S}^{2}\left(i,j\right)W(i,j)$$

where $E_{s}\left(m,n\right)$ is the energy of the region centered on the point (m,n), s represents IR or VIS; Ω(m,n) is the neighborhood window centered on the point (m,n); $A_{s}\left(i,j\right)$ is the low-frequency coefficient of the point (i,j); W(i,j) is the function value of the mask window of the point (i,j). The window function W with a size of 3×3 can be expressed as Equation (10) [32]:

(10) $$W=\frac{1}{16}\left[\begin{array}{ccc} 1 & 2 & 1\\ 2 & 4 & 2\\ 1 & 2 & 1 \end{array}\right]$$

Based on RE, the low-frequency image of IR and VIS are fused by the weighted average RE rule. The weights are shown in Equations (11)–(13).

(11) $$w_{1}=\frac{E_{IR}}{E_{IR}+E_{VIS}}$$

(12) $$w_{2}=1-w_{1}$$

(13) $$f=w_{1}\times IR_{L}+w_{2}\times VIS_{L}$$

where $w_{1}$ and $w_{2}$ are the fusion weights, *f* is the pre-fusion image.

(2) The final fusion based on IFS

**Figure 3:**
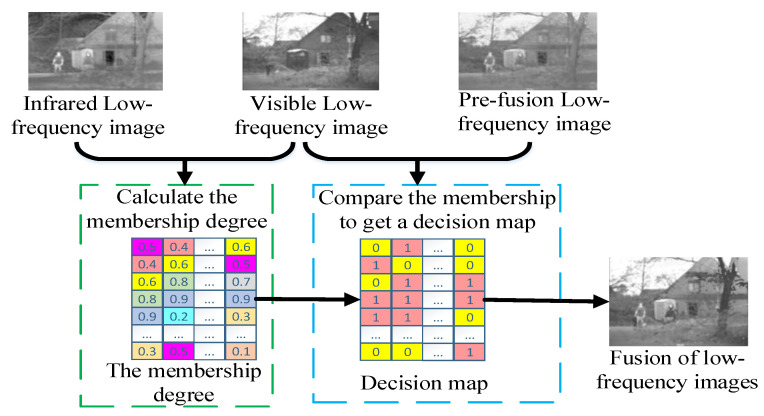
The fusion framework of the detailed layer.

The IFS is introduced to calculate the membership degree of the IR and VIS low-frequency images, and the pre-fusion image is used as the reference to assist the final low-frequency image fusion. Figure 3 shows the low-frequency sub-bands fusion framework.

Gauss membership function is used to represent the membership degree of the coefficients, and the final low-frequency image is fused in accordance with the membership degree after defuzzification. The membership $u_{IR}$ and non-membership $v_{IR}$ of $IR_{L}$ are respectively shown as follows [33]:

(14) $$u_{IR}\left(x,y\right)=\exp\left[\frac{-{\left(IR_{L}\left(x,y\right)-\varepsilon_{IR}\right)}^{2}}{2{\left(k_{1}\sigma_{IR}\right)}^{2}}\right]$$

(15) $$\nu_{IR}\left(x,y\right)=1-\exp\left[\frac{-{\left(IR_{L}\left(x,y\right)-\varepsilon_{IR}\right)}^{2}}{2{\left(k_{2}\sigma_{IR}\right)}^{2}}\right]$$

where $\varepsilon_{IR}$ represents the average value of $IR_{L}$, $\sigma_{IR}$ represents the standard deviation. $k_{1}$ and $k_{2}$ are Gaussian function adjustment parameters. Hesitation degree $\pi_{IR}$ is obtained by $u_{IR}$ and $v_{IR}$. $\pi_{IR}$ is calculated as follows:

(16) $$\pi_{IR}\left(x,y\right)=1-u_{IR}\left(x,y\right)-\nu_{IR}\left(x,y\right)$$

The difference correction method is used to transfer the IFS into FS. The $U_{IR}\left(x,y\right)$ is the membership degree of FS, and which is calculated as below [34]:

(17) $$U_{IR}(x,y)=u_{IR}(x,y)+\pi_{IR}(x,y)\times (0.5+\frac{u_{IR}(x,y)-\nu_{IR}(x,y)}{2})$$

Similarly, the $u_{VIS}$, $v_{VIS}$, $\pi_{VIS}$ and $U_{VIS}$ of VIS low-frequency image can be calculated according to Equations (14)–(17). According to the empirical value, $k_{1}$ and $k_{2}$ are set to 0.8 and 1.2 respectively.

It can be seen from Equations (14)–(17) that the large gray value of the pixel corresponds to the small membership value. Therefore, in IR images, the targets own the smaller membership value. In VIS images, the background and texture features own the smaller membership value. The membership degree can be used to determine which valuable pixels can be integrated into the final resulting image. Using the pre-fusion image f as the reference image, the $U_{IR}$ and $U_{VIS}$ are compared to get the decision map to generate the final fused image $F_{L}$. The specific fusion rule is defined as below:

(18) $$F_{L}=\{\begin{array}{cc} VIS_{L}, & U_{IR}\geq U_{VIS}\\ f, & U_{IR}<U_{VIS} \end{array}$$

### 3.3. The Rule for High-Frequency Components

Different from the low-frequency sub-bands, the high-frequency sub-bands are usually used to reflect the texture and contour information of the source images. The edges and contours are important information carrying points used to display the visual structure of the image. Edges and contours often correspond to the pixels with a sharp decrease in brightness information. Therefore, we adopt the max-absolute fusion rule to fuse the high-frequency sub-bands to get rich texture information. The fusion rule can be described as follows:

(19) $$F_{H}^{j,k}=\{\begin{array}{cc} IR_{H}^{j,k}, & \left|IR_{H}^{j,k}\right|>\left|VIS_{H}^{j,k}\right|\\ VIS_{H}^{j,k}, & \left|IR_{H}^{j,k}\right|\leq \left|VIS_{H}^{j,k}\right| \end{array}$$

where $F_{H}^{j,k}$ is the final fusion results of the high-frequency sub-bands.

### 3.4. NSST Reconstruction

The fused image F is reconstructed by the inverse NSST transform according to Equation (20).

(20) $$F=NSST\_REC(F_{L},F_{H}^{j,k})$$

where the NSST_REC(·) represents the inverse NSST transform function; F is the output image.

The proposed RE-IFS-NSST method is summarized in Algorithm 1.
**Algorithm 1.** The proposed RE-IFS-NSST fusion algorithm.Input: Infrared image (IR), Visible image (VIS)Out: Fused image (*F*).The IR and VIS are decomposed by NSST to obtain the coefficients of high- and low-frequency components $\left\{IR_{L},IR_{H}^{j,k}\right\}$ and $\left\{VIS_{L},VIS_{H}^{j,k}\right\}$ according to Equations (7) and (8);Calculate the fusion weight of the low-frequency images $IR_{L}$ and $VIS_{L}$ according to the Equations (6)–(11) to get the pre-fusion result;According to Equations (12)–(16) the final fusion result of low-frequency part $F_{L}$ is obtained;Calculate the $\left\{IR_{H}^{j,k},VIS_{H}^{j,k}\right\}$ membership degree according to Equation (17) to obtain the $F_{H}^{j,k}$;Reconstruct $\left\{F_{L},F_{H}^{j,k}\right\}$ to get the final fusion result F according to Equation (18).

## 4. Experimental Results

### 4.1. Datasets

In order to test the effectiveness of the proposed method, the experiments are conducted on two public datasets, TNO Image Fusion Dataset and RoadScene Dataset, which are widely used in the field of IR and VIS image fusion. We chose 6 sets of IR and VIS images in TNO dataset and 5 sets of IR and VIS images in RoadScene dataset. All the images pairs can be downloaded from: https://github.com (accessed on 15 July 2021).

### 4.2. Experimental Setting

In order to test the practicability and effectiveness of the proposed method, we set up two groups of experiments. The first group compares the proposed method with RE-NSST and IFS-NSST methods, and the second group compares the proposed method with the other nine advanced fusion methods which are FPDE [35] (fourth-order partial differential equations), VSM [36] (visual saliency map), Bala [37] (Bala fuzzy sets), Gauss [34] (Gauss fuzzy sets), DRTV [38] (Different Resolutions via Total Variation Model), LATLRR [39] (latent Low-Rank Representation), SR [40] (sparse regularization), MDLatLRR [41] (decomposition method based on latent low-rank representation) and RFN-Nest [42] (residual fusion network).

The experimental parameters are set as follows:

(1) The computer is configured as 2.6 Hz Intel Core CPU and 4GB memory, and all experimental codes run on the Matlab2017 platform.
(2) In the proposed method, the ‘maxflat’ is chosen as the pyramid filter. The numbers of decomposition level and directions are 3 and {16,16,16}, respectively.
(3) In the RE-NSST and IFS-NSST methods, the parameters of NSST are the same as that of the proposed method. The calculation of RE and IFS are the same as that of the proposed method.
(4) In Bala and Gauss methods, the ‘9-7′ and ‘pkva’ are chosen as the pyramid filter and the directional filter respectively, and the decomposition scale is 3.
(5) In the MDLatLRR method, the decomposition level selection 2.
(6) The parameters of the other 9 methods are set following the best parameter setting reported in the corresponding papers.

### 4.3. Quantitative Evaluation

In this section, six types of quantitative evaluation indexes are introduced to evaluate the performance of every algorithm objectively, including E, AG, MI, CE, SPD, and PSNR. Among the indexes, the E and AG are non-reference indexes; the MI, CE, SPD, and PSNR are reference-based indexes. MI and CE utilize IR and VIS images as the reference to calculate the similarity and difference between the source image and the fused image. SPD and PSNR use the VIS image as the reference to reflect the interference information from the VIS image into the fused image. To comprehensively evaluate the fusion performance from different aspects, we employ both the reference-based and no-reference indexes in this study.

(1) Entropy (E) [43]

E describes the average amount of information of the source image, and it is calculated using Equation (21):

(21) $$E=-\sum_{i=1}^{m}p_{i}\log_{2}p_{i}$$

where L represents the total gray level, $p_{i}$ is the probability of the gray value i.

(2) Average Gradient (AG) [43]

AG reflects the micro-details contrast and texture features variation of the fused image. It can be expressed as below:

(22) $$AG=\frac{1}{M\times N}\sum_{m=1}^{M}\sum_{n=1}^{N}\sqrt{\frac{\Delta F_{x}^{2}(m,n)+\Delta F_{y}^{2}(m,n)}{2}}$$

where the area with the pixel (m,n) as the center and size M×N, $\Delta F_{x}$ and $\Delta F_{y}$ are the difference in two directions of the fused image F.

(3) Mutual Information (MI) [44]

MI reflects the amount of information transferred from the source images to fused image, which can be calculated as follows:

(23) $$MI_{X,F}=\sum_{i=1}^{L}\sum_{j=1}^{L}P_{X,F}(i,j)\log_{2}\left(\frac{P_{X,F}\left(i,j\right)}{P_{X}(i)P_{F}(j)}\right)$$

(24) $$MI=MI_{IR,F}+MI_{VIS,F}$$

where $P_{X}$ and $P_{F}$ respectively represent the gray distribution of the source images and the fused image, and $P_{X,F}$ are the joint probability distribution density. The sum of $MI_{A,F}$ and $MI_{B,F}$ denotes the mutual information value.

(4) Cross Entropy (CE) [44]

CE reflects the different degree of gray distribution between fusion image and source images, which is defined as below:

(25) $$CE(IR,F)=\sum_{i=1}^{L}p_{i}\log_{2}\left(\frac{p_{i}}{q_{i}}\right)$$

(26) $$CE(VIS,F)=\sum_{i=1}^{L}v_{i}\log_{2}\left(\frac{v_{i}}{q_{i}}\right)$$

(27) $$CE(IR,VIS,F)=\sqrt{\frac{CE^{2}\left(IR,F\right)+CE^{2}\left(VIS,F\right)}{2}}$$

where L is gray levels, $p_{i}$, $\nu_{i}$ and $q_{i}$ are the probability of the detected pixel with gray value i appearing in the IR, VIS, and the fused images.

(5) Spectral Distortion (SPD) [45]

SPD reflects the degree of color distortion between the fused image and the VIS image, the expression is shown in Equation (28):

(28) $$SPD=\frac{1}{M\times N}\sum_{i=1}^{M}\sum_{n=1}^{N}\left|F\left(i,j\right)-A\left(i,j\right)\right|$$

where F(i,j) and VIS(i,j) represent the gray values of the fused image and the VIS image at (i,j) respectively.

(6) Peak signal to noise ratio (PSNR) [44]

PSNR is mainly used to measure the ratio between effective information and noise of image, and it can illustrate whether the image is distorted. It can be given as below:

(29) $$MSE=\frac{1}{M\times N}{\sum_{i=1}^{M}\sum_{j=1}^{N}\left(F\left(i,j\right)-VIS\left(i,j\right)\right)}^{2}$$

(30) $$PSNR=10\mathrm{lg}\frac{Z^{2}}{MSE}$$

where F(i,j) and VIS(i,j) represent the gray values of the fused image and the source image at (i,j) respectively. MSE is the mean square error, and it reflects the degree of difference between variables. Z represents the difference between the maximum and minimum gray value of the source image.

### 4.4. Fusion Results on the TNO Dataset

#### 4.4.1. Comparison with RE-NSST and IFS-NSST Methods

**Figure 4:**
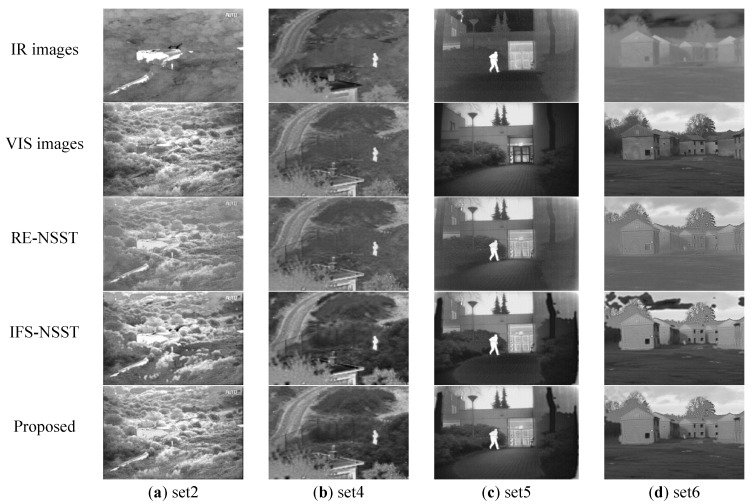
The fusion results in TNO Dataset. The first and second rows are the IR and VIS images. From the third to the fifth row are the fusion results of RE-NSST, IFS-NSST and Proposed methods on 4 sets of the source images.

In the first group of simulation experiments, we firstly compare three methods, the RE-NSST, IFS-NSST, and proposed methods. The qualitative comparison results are shown in Figure 4.

As shown in Figure 4, the three methods can fuse the source images, but the results are different. The RE-NSST method can achieve relatively obvious targets, but the fused images have the problems of low contrast and image blurring. The fused images of the IFS-NSST method have higher contrast and more detailed information, but the problems of false contour and block effects are inevitable. The fused images have poor visual effects. Compare with the RE-NSST and IFS-NSST methods, the proposed method can extract the complete infrared targets and continuous and clear edge details. The fused images are more suitable for human-eye observation.

The quantitative comparison results are shown in Table 1. We use red and blue to represent the best and second-best results respectively. For the E, AG, MI, and PSNR, the large values mean better performance. For the CE and SPD, the low values mean better fusion performance. Except for AG, the proposed method is superior to RE-NSST and IFS-NSST in other parameters, which means that the proposed method has the best comprehensive performance.

#### 4.4.2. Comparison with the State-of-the-Art Methods

**Figure 5:**
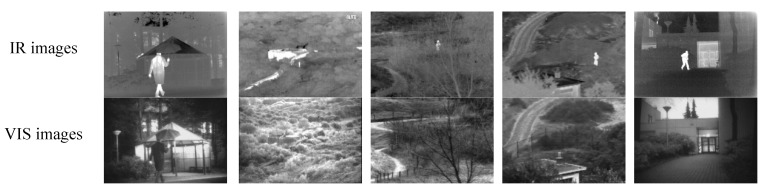

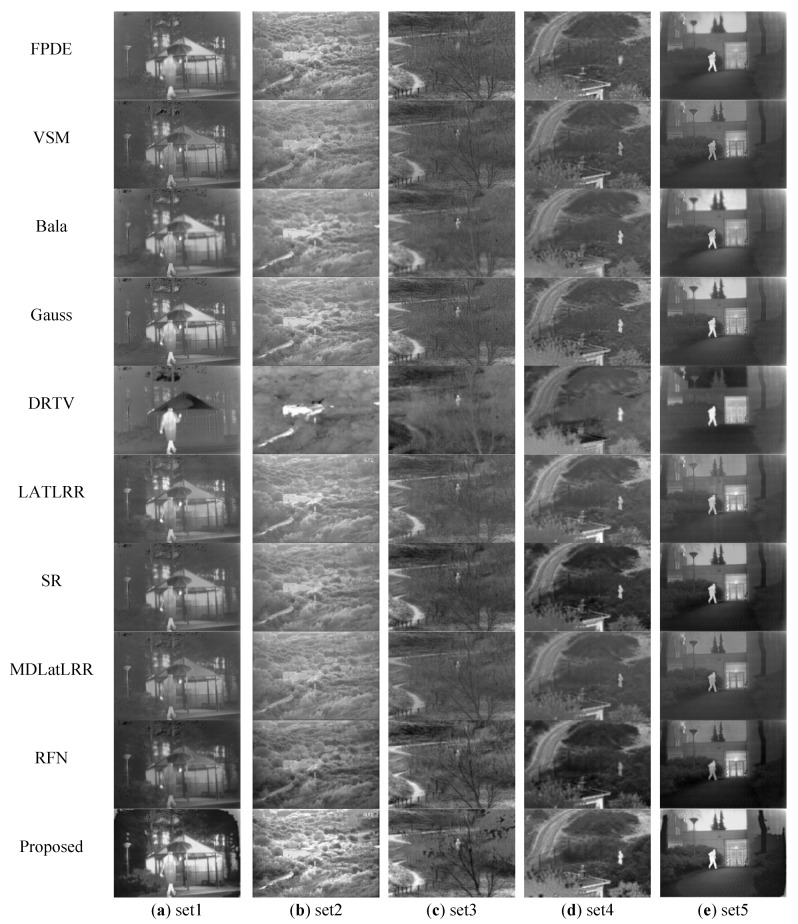
Results on TNO dataset. From the first row to the last row are IR images, VIS images, the fusion results of FPDE, VSM, Bala, Gauss, DRTV, LATLRR, SR, MDLatLRR, RFN, and the proposed methods.

In the second group of the experiment, we compare the proposed method with other advanced methods. Figure 5 shows the qualitative fusion results on the TNO dataset. As shown in Figure 5, the compared Bala method can achieve complete infrared targets, but the background is blurring. The Gauss method can preserve more texture features (such as the edges of the window and road). However, the fused images have the problems of obvious block effects. The FPDE lost the detailed edge information (e.g., roads, trees, shrubs, windows, or street lights) and make the scene recognition difficult. The infrared targets information is highlighted in the fusion image of DRTV, but the background is blurred and the edges information is lost seriously. VSM, LATLRR, MDLatRR, SR, and RFN can achieve relatively rich details, but the brightness of the infrared targets is low. Compared with the nine methods, the proposed method can obtain better image performance. The fused images have more obvious infrared targets and abundant background and detailed information, which are more suitable for the human visual system.

**Figure 6:**
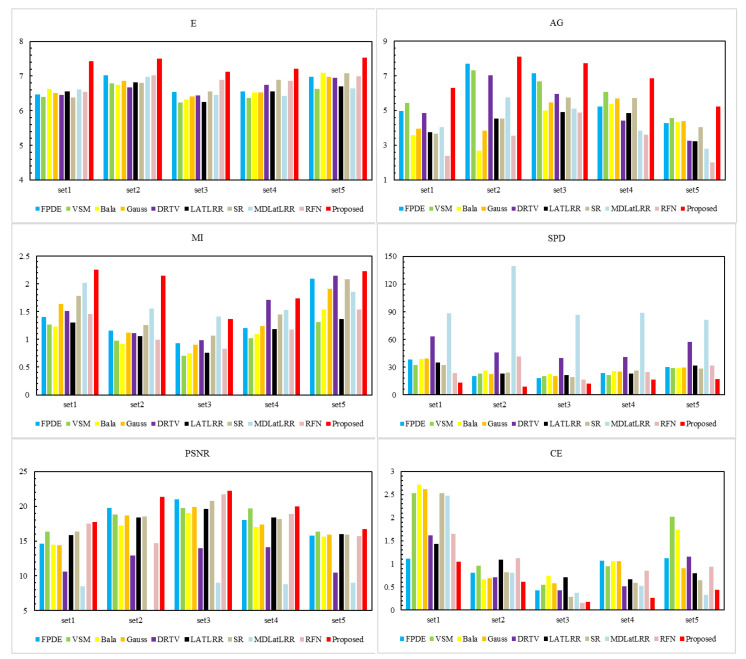
Comparison results of six evaluation parameters. The nine methods, i.e., FPDE, VSM, Bala, Gauss, DRTV, LATLRR, SR, MDLatLRR, RFN are compared with the proposed method.

Figure 6 displays the quantitative fusion results of the ten methods. The results show that our proposed method gets the best performance on 4 objective evaluation metrics (E, AG, SPD, PSNR) and the second-best performance on 2 objective evaluation metrics (MI, CE). The differences between the best MI and CE are small, superior to other compared methods. 

#### 4.4.3. Analysis

**Figure 7:**
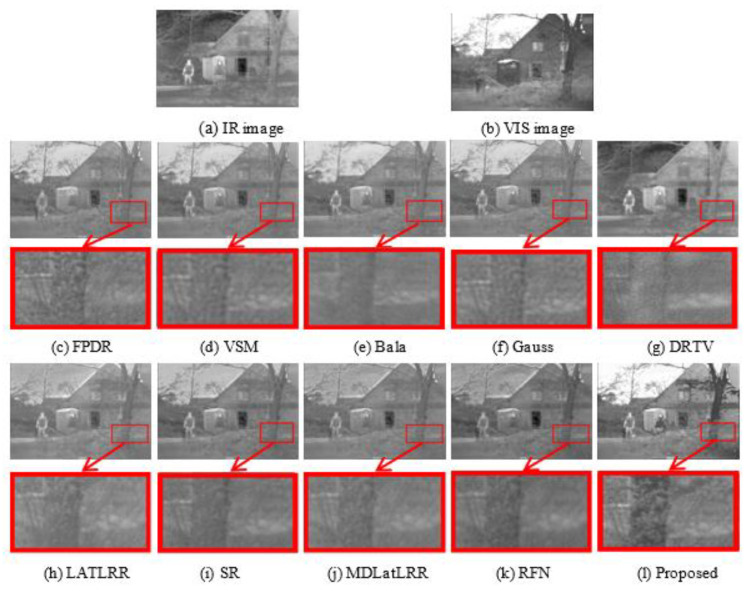
Fusion results on ‘2_Men in front of house’ image. From (**c**–**l**) are the fusion result of the ten methods.

We use the ‘2_Men in front of house’ image as the example to further illustrate the superiority of this algorithm. We enlarge the region contained in the red rectangle in the fused image by the same multiple. The quantitative comparison results of all ten methods are shown in Figure 7.

The magnifying detailed images illustrate that the proposed method has the following advantages:

(1) The proposed method can transfer more detailed textures features of shrub and tree to the resulting image.
(2) The proposed method can preserve obvious infrared targets information in the resulting image.
(3) The proposed method can improve the image contrast and brightness.

The objective evaluation results of ten methods on ‘2_Men in front of house’ image are shown in Table 2. Table 2 illustrates that the proposed method obtains the best results on the 6 parameters. In general, the proposed method performs better than other compared methods.

### 4.5. Fusion Results on the RoadScene Dataset

**Figure 8:**
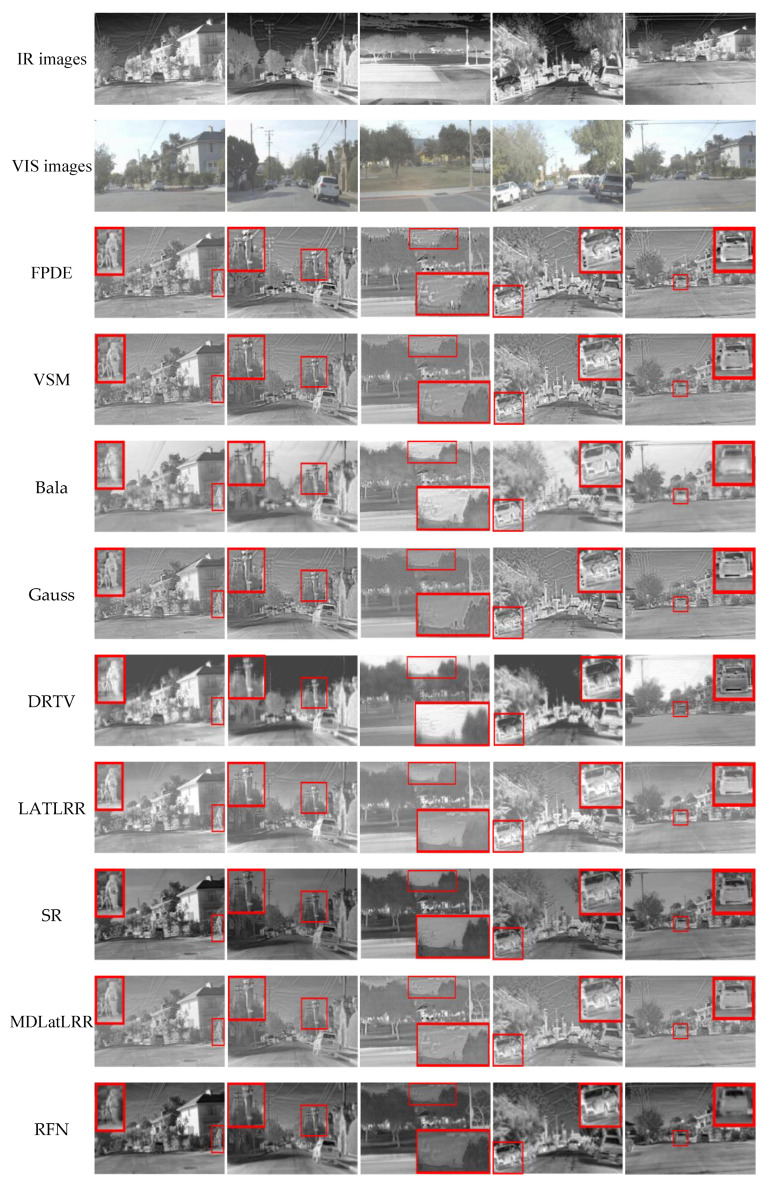

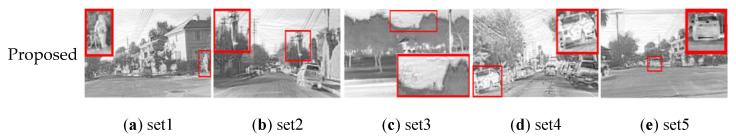
Results on the RoadScene dataset. From the first row to the last are IR images, VIS images, the fusion results of FPDE, VSM, Bala, Gauss, DRTV, LATLRR, SR, MDLatLRR, RFN, and the proposed method.

To further evaluate the applicability of the proposed method, we conduct experiments on the RoadScene dataset. The images on the RoadScene datasets contain rich road traffic scenes such as vehicles, pedestrians, and roads. The VIS images in the RoadScene dataset are color images. In the fusion experiment, they are first converted to gray images. We choose 5 pairs of typical images to carry on the comparison experiments. We enlarge the details of the region contained in the red rectangle in the fused images. The comparison results are shown in Figure 8.

Figure 8 shows that the fusion image obtained by SR, and RFN methods are darker than other images. The infrared targets of FPDE and VSM methods are not prominent. Although the DRTV method can get an obvious target, it can’t identify the background texture features. The Bala method loses the image detail information. The fusion images of LATLRR and MDLatLRR methods have lower contrast. The fused images of the proposed method can achieve plentiful detailed texture features and apparent infrared targets. The qualitative results show that the proposed method can obtain better fusion performance, which is also applicable for the RoadScene dataset.

**Figure 9:**
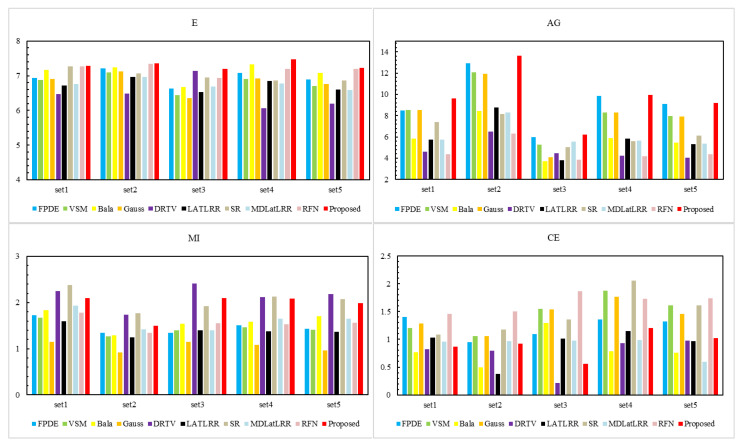

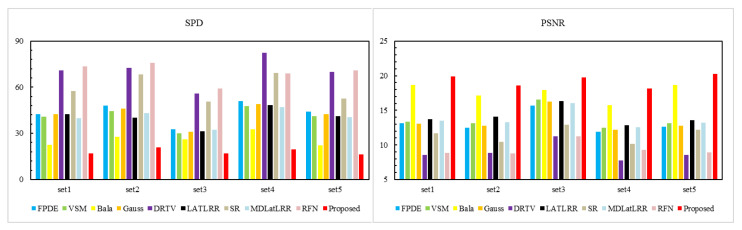
Quantitative results of six evaluation parameters. The nine methods, i.e., FPDE, VSM, Bala, Gauss, DRTV, LATLRR, SR, MDLatLRR, RFN are compared with the proposed method.

Figure 9 shows the quantitative data results on the RoadScene dataset of all the fusion methods. The results show that our proposed method performs best on 4 objective evaluation metrics (E, AG, SPD, PSNR) and the second-best on the other two metrics (MI and CE). Our proposed method achieves a lower value of MI and a higher value of CE. The reason is maybe that we discard some useless interference information, such as noise information, which existed since the image was collected by the RoadScene dataset. Therefore, the similarity between the fusion image and the source image is relatively small, resulting in lower MI and higher CE values.

The results on the RoadScene dataset are basically consistent with those on the TNO dataset. Qualitative and quantitative experimental results show that the proposed method can generate fused images with prominent targets and abundant details, which is more appropriate for the human visual system.

### 4.6. The Computational Complexity Analysis

In order to analyze the computational complexity, we calculate the running time of different methods when fusing the above image pairs of TNO and RoadScene datasets. All experiments were performed under the same conditions. The results of the running time are shown in Table 3 and Table 4. The best and second-best running time is displayed in red and blue respectively. The results of the proposed method are shown in bold.

According to Table 3 and Table 4, the DRTV shows the best calculation efficiency than other fusion methods, but the fusion performance is not the most satisfactory. The calculation complexity of VSM is lower on TNO dataset, but it cannot get the same results on the RoadScene dataset. LATLRR and MDLatLRR are relatively long. The running time of the MDLatLRR method is closely related to the decomposition level. As the number of decomposition levels increases, the running time of the algorithm becomes increasingly longer. Although the proposed method cannot obtain the lowest running time, the fusion quality is superior to other methods. At the same time, the method is more stable when dealing with different datasets.

## 5. Conclusions

In this paper, we present a new fusion method employing RE and IFS in the NSST domain for IR and VIS images. Thanks to the RE-IFS-NSST fusion strategy, the fusion image can have apparent infrared targets information and more plentiful texture features simultaneously. We conduct the experiments on the two public datasets, and six evaluation indexes are used to test the performance of the presented method. Quantitative results show that the proposed method is superior to nine other methods. Compared with the best results of the nine methods, the E, AG, PSNR, and SPD of this method on the TNO dataset are increased by 7.2%, 12.9%, 5.5%, and 92.3%, respectively; the same four parameters on the RoadScene dataset are increased by 7.4%, 1.5%, 13.5%, and 25.7%. The qualitative results demonstrate that the fused images have better fusion quality, and they are more consistent with the human visual system. The proposed method has a good application in the target detection field, such as in the military, medical diagnosis, target tracking, etc.

## Figures and Tables

**Table 1:** Quantitative comparison results of RE-NSST, IFS-NSST, and proposed methods.

Pictures	Algorithm	E	AG	MI	CE	SPD	PSNR
set2	RE-NSST	7.0231	7.8391	1.5491	**0.4003**	20.2137	**18.6581**
	IFS-NSST	**7.4985**	**8.2866**	**1.6744**	0.4143	**17.9667**	17.4212
	**Proposed**	**7.5003**	**8.1158**	**2.1463**	**0.3881**	**9.1239**	**21.3318**
set4	RE-NSST	6.6502	6.6127	1.3885	1.1583	27.0561	16.3921
	IFS-NSST	**7.1960**	**6.9817**	**1.5673**	**0.4401**	**23.0697**	**16.2754**
	**Proposed**	**7.2065**	**6.8724**	**1.7395**	**0.2678**	**16.6751**	**17.9727**
set5	RE-NSST	7.1367	5.0369	2.1408	0.8499	30.3678	14.9696
	IFS-NSST	**7.4193**	**5.2169**	**2.1355**	**0.7068**	**22.5062**	**15.6012**
	**Proposed**	**7.5317**	**5.2223**	**2.2287**	**0.4438**	**17.1070**	**16.7450**
set6	RE-NSST	6.7152	5.1251	2.3817	2.5125	30.7200	**17.3706**
	IFS-NSST	**7.1657**	**5.6524**	**2.7181**	**1.4545**	**23.0795**	14.9469
	**Proposed**	**7.1764**	**5.2818**	**3.0924**	**1.2185**	**10.6998**	**22.1216**

**Table 2:** Quantitative comparison results of the ten methods on ‘2_Men in front of house’ image.

Algorithm	E	AG	MI	CE	SPD	PSNR
FPDE	6.6385	5.1961	1.5334	1.1573	25.8568	17.8237
VSM	6.5374	5.0431	1.1345	1.5274	30.3846	15.9714
Bala	6.7515	2.5025	1.3897	0.6556	28.1342	16.8945
Gauss	6.7573	3.3446	1.4076	1.5874	27.1725	17.3515
DRTV	7.0767	4.9648	1.9273	0.8521	60.3667	10.0039
LATLRR	6.6468	3.3042	1.1525	1.2658	31.8783	15.9835
SR	6.6610	3.4351	1.7760	1.6768	27.7083	17.1956
MDLatLRR	6.6913	3.9260	1.8946	0.4422	134.4224	5.3021
RFN	6.6424	2.7265	1.1956	1.4660	30.4971	15.5273
Proposed	7.1322	5.6427	2.0628	0.2455	14.5461	19.2055

**Table 3:** Running time T (s) of the ten methods on the TNO dataset.

Images	FPDE	VSM	Bala	Gauss	DRTV	LATLRR	SR	MDLatLRR	RFN	Proposed
set1	11.0281	**2.1048**	32.5587	33.2193	**0.8448**	105.9846	6.0897	150.6458	10.6317	**3.1674**
set2	18.6852	**3.6156**	49.1194	49.3599	**1.3805**	111.3046	10.266	186.3398	11.4100	**4.8280**
set3	10.2954	**2.2599**	31.4941	31.0160	**0.8172**	99.6353	5.9340	180.6707	11.9672	**3.1971**
set4	1.7641	**0.8080**	22.3608	19.6283	**0.2517**	33.6849	1.7168	80.0596	12.7350	**1.3497**
set5	10.8291	**2.1046**	32.4518	32.0341	**0.8023**	106.767	6.3531	161.4593	13.6248	**3.1962**

**Table 4:** Running time T (s) of the ten methods on the RoadScene dataset.

Images	FPDE	VSM	Bala	Gauss	DRTV	LATLRR	SR	MDLatLRR	RFN	Proposed
set1	5.3412	**2.0977**	36.0400	37.9651	**0.4194**	104.1814	3.1564	153.4153	9.7678	2.3402
set2	2.6183	3.0216	17.0855	19.6853	**0.2962**	75.0036	1.7318	95.97211	10.8458	**1.5268**
set3	7.6224	4.6006	24.3446	25.7651	**0.5192**	112.2796	3.1698	192.0942	11.7744	**1.9909**
set4	2.7065	5.3109	14.0975	15.7018	**0.2460**	55.0571	1.5370	76.53124	12.1473	**1.2707**
set5	6.1202	6.8466	23.5806	25.0931	**0.4660**	103.0710	3.0122	162.6771	13.0335	**1.9509**

## Data Availability

Not applicable.
